# Development, Validation and Comparison of Artificial Neural Network Models and Logistic Regression Models Predicting Survival of Unresectable Pancreatic Cancer

**DOI:** 10.3389/fbioe.2020.00196

**Published:** 2020-03-13

**Authors:** Zhou Tong, Yu Liu, Hongtao Ma, Jindi Zhang, Bo Lin, Xuanwen Bao, Xiaoting Xu, Changhao Gu, Yi Zheng, Lulu Liu, Weijia Fang, Shuiguang Deng, Peng Zhao

**Affiliations:** ^1^Department of Medical Oncology, The First Affiliated Hospital, Zhejiang University School of Medicine, Hangzhou, China; ^2^College of Computer Science and Technology, Zhejiang University, Hangzhou, China; ^3^Technical University Munich (TUM), Munich, Germany; ^4^Department of Medical Oncology, Tai He People's Hospital, Fuyang, China; ^5^Internal Medicine, Cangnan Traditional Chinese Medicine Hospital, Wenzhou, China; ^6^Zhejiang Provincial Key Laboratory of Pancreatic Disease, Hangzhou, China

**Keywords:** artificial neural network, logistic regression, unresectable pancreatic cancer, survival, prognosis

## Abstract

**Background:** Prediction models for the overall survival of pancreatic cancer remain unsatisfactory. We aimed to explore artificial neural networks (ANNs) modeling to predict the survival of unresectable pancreatic cancer patients.

**Methods:** Thirty-two clinical parameters were collected from 221 unresectable pancreatic cancer patients, and their prognostic ability was evaluated using univariate and multivariate logistic regression. ANN and logistic regression (LR) models were developed on a training group (168 patients), and the area under the ROC curve (AUC) was used for comparison of the ANN and LR models. The models were further tested on the testing group (53 patients), and k-statistics were used for accuracy comparison.

**Results:** We built three ANN models, based on 3, 7, and 32 basic features, to predict 8 month survival. All 3 ANN models showed better performance, with AUCs significantly higher than those from the respective LR models (0.811 vs. 0.680, 0.844 vs. 0.722, 0.921 vs. 0.849, all *p* < 0.05). The ability of the ANN models to discriminate 8 month survival with higher accuracy than the respective LR models was further confirmed in 53 consecutive patients.

**Conclusion:** We developed ANN models predicting the 8 month survival of unresectable pancreatic cancer patients. These models may help to optimize personalized patient management.

## Introduction

Pancreatic cancer is one of the leading causes of cancer-related mortality worldwide (Ferlay et al., 2015). Most patients present with few specific symptoms and are diagnosed at an advanced stage. Despite the development of surgical techniques, radiotherapy and chemotherapy, the prognosis of pancreatic cancer is dismal (Hidalgo et al., 2015). In most cases, the disease itself leads to the patients' short survival time, and treatment rarely achieves cure, although some patients achieve remissions lasting several years (Kuhlmann et al., 2004; Cress et al., 2006; Bradley, 2008). Given that life expectancy is relatively short, even in the face of optimal treatment, doctors must weigh the potential survival benefits with the potential impact of treatment complications on patients' quality of life.

Different predictive evaluation systems or risk scores have been developed for decision-making, including perioperative mortality risk (Are et al., 2009), post-surgery complications (Braga et al., 2011) and survival prediction (Miura et al., 2014; Dasari et al., 2016). Survival prediction models help doctors make appropriate recommendations for the most suitable treatment option, thus maximizing the survival benefit. In addition, proper and uniform prediction models can facilitate more accurate enrolment in clinical trials. Nevertheless, current options to predict overall survival remain unsatisfying. The TNM classification developed by the American Joint Committee on Cancer has been used to estimate the prognosis of cancer. However, there are different prognoses in pancreatic cancer patients whose TNM stages are similar (Xu et al., 2017). Previous clinical research has shown the predictive effect of clinical pathological biomarkers such as tumor heterogeneity, main vessel invasion, and complexity at the genomic, epigenetic, and metabolic levels in patients with pancreatic cancer (Kleeff et al., 2016; Neoptolemos et al., 2018; Naito et al., 2019). However, these predictive biomarkers still have many limitations. Additional reliable prognostic indicators are urgently needed.

Artificial neural networks (ANNs), a commonly used method of machine learning, work in a non-linear mode and model a biological neural system both structurally and functionally (Cucchetti et al., 2010). In addition to its application in the field of computer engineering, ANN modeling emerges as a potential useful tool for projecting clinical outcomes (Penny and Frost, 1996). Many clinical studies have compared the predictive power of ANN models with logistic regression (LR) models and have shown ANNs to have better performance (Hanai et al., 2003; Ghoshal and Das, 2008). A systemic review showed an increase in the benefit of ANNs over existing statistics in healthcare provision (Lisboa and Taktak, 2006). However, few studies have compared the performance of ANN with LR in the field of pancreatic cancer.

In our study, we aimed to explore possible prognostic indicators for unresectable pancreatic cancer on the basis of clinical and radiological variables and investigate the diagnostic accuracy of these two methodologies (LR, ANN) in predicting overall survival. The performance of the ANN and logistic regression models were validated externally using a different data set.

## Materials and Methods

### Patients

We retrospectively reviewed 221 cases of unresectable pancreatic cancer registered between May 2010 and December 2018 at the First Affiliated Hospital of Zhejiang University. Taking January 2018 as the dividing point, patients were classified into two groups: 168 patients were used as a training dataset, and 53 patients were used as an independent validation dataset. The inclusion criteria for patients were as follows: (i) patients were histologically confirmed adenocarcinoma of the pancreas; (ii) resectability status were evaluated as unresectable according to the Pancreatic Adenocarcinoma NCCN Guidelines; (iii) patients were ≥18 years of age and had a Eastern Cooperative Oncology Group (ECOG) score 0–2; (iv) patients had adequate hematologic, hepatic, and renal function before treatment; (v) Complete clinical imaging data and biochemical data 2 weeks before chemotherapy and survival data were available. The exclusion criteria were: (i) patients received prior chemotherapy or surgery; (ii) recurrent pancreatic cancer. The study followed the international and national regulations in accordance with the Declaration of Helsinki and was approved by the ethics committee of the First Affiliated Hospital, Zhejiang University School of Medicine. The following clinical and biochemical data were collected before the patient received chemotherapy: age, sex, main vascular invasion (celiac axis, superior mesenteric artery, common hepatic artery), clinical TNM staging, metastasis (including retroperitoneal lymph node, liver, lung and peritoneum), ascites, size of the largest tumor in the pancreas and liver, tumor position in the pancreas, stomach invasion, duodenum invasion, liver metastasis number, carcinoembryonic antigen (CEA), carbohydrate antigen 199 (CA199), albumin-to-globulin ratio (AGR), alanine transaminase (ALT), aspartate transaminase (AST), creatinine, total bilirubin, direct bilirubin, indirect bilirubin, haemoglobulin, neutrophil/lymphocyte ratio, platelet/lymphocyte ratio, hepatitis B virus, and white blood cell (WBC) count. Pancreatic tumor or metastatic lesions directly invading stomach was defined as stomach invasion which was diagnosed based on patients' imaging, according to pancreatic ductal adenocarcinoma radiology reporting template (Al-Hawary et al., 2014). Progression-free survival (PFS), overall survival (OS), and chemotherapy regimen were recorded. All patients underwent primary palliative chemotherapy. TNM staging was adopted according to the NCCN Guidelines (version 1. 2019) for pancreatic cancer. The number of tumors, size of the largest tumor (cm), tumor position, and metastasis or invasion organs were defined for all patients on the basis of the CT scan or MRI.

### Follow-Up

Patients were followed by outpatient clinics or phone calls until September 2019. These follow-ups were conducted at 3 month intervals. OS was defined as the number of months from the date of diagnosis to the date of death or the date of last follow-up. PFS was defined as the number of months from the date of diagnosis to the date of identification of disease progression. In this study, the median follow-up duration was 9 (range 3–36) months.

### Statistical Analysis

All patient characteristics in the training and testing groups were compared. Continuous variables with parametric distributions were evaluated by *t*-test. Categorical variables were evaluated by χ^2^-test (or Fisher's exact test, if appropriate). OS was estimated using the Kaplan–Meier method. The association of the baseline parameters with 8 month survival was assessed using univariate logistic regression analyses, and those with *p* < 0.05 were entered into multivariate logistic regression analyses. Significantly skewed continuous variables (CEA, CA199, ALT, AST, total bilirubin, direct bilirubin, indirect bilirubin, haemoglobulin, the neutrophil/lymphocyte ratio, the platelet/lymphocyte ratio, and WBC count) were normalized by logarithmic transformation. The violin plot was generated using the Python (version 3.7.5) seaborn library.

### Development of the Logistic Regression Models

In the training set of 168 patients, variables found to be significantly related to 8 month survival in the multivariate analysis and univariate analysis were entered into logistic regression models 1 and 2, respectively. All 32 variables were entered into logistic regression model 3. A total of 168 patients in the training group were selected to train the logistic regression model, and the remaining 53 patients were used for testing. Logistic regression is a predictive linear model that can be used to predict the causality relationship between a dependent binary variable and one or more independent variables. The formula for logistic regression can be simply presented in linear algebra terms as *Y* = *A*^*T*^*X* + *b*., where Y is the output of our model and X is the input. Both A and b are parameters to be learned from training data. The learned parameter A can be interpreted as the relative importance of each factor in the survival of the patient. Our logistic regression models were built using the Python scikit-learn library.

### Development of the Artificial Neural Network Models

In the training group (*n* = 168), 133 (80%) patients were randomly selected to train the network, while 35(20%) for cross validation. Cross-validation was necessary for our neural networks to learn general predictive characteristics rather than memorizing the idiosyncrasies of the training data, which played a role in helping assisting model building, including stopping network training and to avoiding over-fitting.

**Figure 1:**
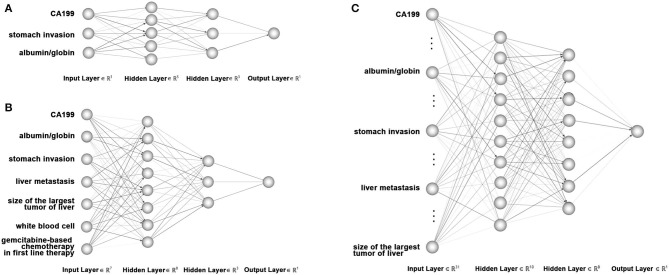
Diagram of artificial neural network models used to predict 8 month survival of unresectable pancreatic cancer. **(A)** Artificial neural network model with 3 input nodes: stomach invasion, AGR and CA199. **(B)** Artificial neural network with 7 input nodes: liver metastasis, stomach invasion, size of the largest tumor of the liver, CA199, AGR, white blood cell count, and gemcitabine-based chemotherapy as the first-line therapy. **(C)** Artificial neural network with 32 input nodes. The output nodes of the three ANN models were 8 month survival.

In the training set of 168 patients, variables found to be significantly related to 8 month survival in the multivariate analysis and univariate analysis were entered into ANN models 1 and 2, respectively. All 32 variables were entered into ANN model 3. A total of 168 patients in the training group were selected to train the network, and the remaining 53 patients were used for testing. Our artificial neural network was built using the PyTorch framework. The search space of network configuration was based empirically on the number of features and the quantity of our available data. And then grid search was conducted to search the best network configuration based on the criteria of our cross-validation group (Bergstra and Bengio, 2012). We have tried three layers or five or more layers, all resulting dissatisfied or overfitting and the best performance was achieved with four layers based on computer experiments. So we built a four-layer feedforward neural network with 3 input nodes in the input layer, 5 and 3 nodes in the first and second hidden layers, respectively, and one output neuron in model 1; 7 input nodes, 8 and 3 neurons in two hidden layers, and one output neuron in model 2; and 32 input nodes, 10 and 8 neurons in two hidden layers, and one output neuron in model 3. Figure 1 shows the diagrams of ANN models 1–3. The selection strategy was stratified sampling, which guaranteed that the ratios of positive and negative samples in both groups were equal. An early-stop strategy, which stops the training process when the performance of cross-validation no longer improves, was applied in the training of our neural networks.

### Assessment of the Diagnostic Accuracy of the Models

The accuracy of the ANN and logistic regression models in predicting 8 month OS were compared using receiver operating characteristic (ROC) curve analysis, positive predictive values (PPV), and positive likelihood ratios (PLR). The performance parameters were calculated by the following formulas: sensitivity: TP/(TP+FN), specificity: TN/(FP+TN), accuracy: (TP+TN)/(P+N), positive predictive value: TP/(TP+FP), negative predictive value: TN/(TN+FN), and positive likelihood ratio = sensitivity/(1-specificity), where TP is true positive, FN is false negative, FP is false positive, TN is true negative, P is positive, and N is negative. The Hanley–McNeil method was used to compare ROC curves. The predictions of both the ANN and logistic regression models in the testing group of 53 patients were reported using Cohen's k coefficient using the formula: [Pr(a)–Pr(e)]/[1–Pr(e)]; Pr(a) is the relative observed agreement, and Pr(e) is the proportion of agreement expected to occur by chance alone (Landis and Koch, 1977). Statistical and ROC analyses were performed by MedCalc 7.2.1.0 (MedCalc software, Mariakerke, Belgium).

## Results

### Patient Demographics

**Figure 2:**
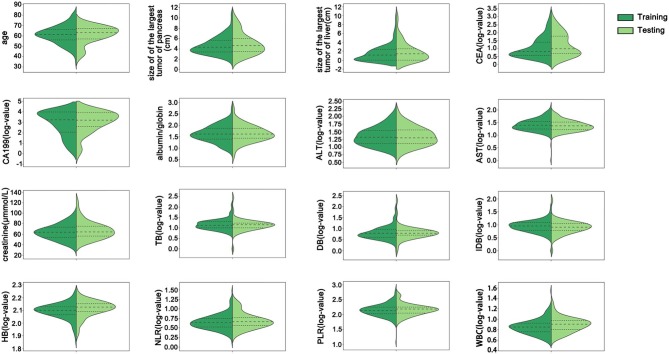
The distribution of all continuous variables in the training and testing groups. There were no significant differences between the training and testing groups in any continuous variables. CEA, carcinoembryonic antigen; CA199, carbohydrate antigen 199; ALT, alanine transaminase; AST, aspartate transaminase; TB, total bilirubin; DB, direct bilirubin; IDB, indirect bilirubin; HB, hemoglobin; NLR, neutrophil/lymphocyte ratio; PLR, platelet/lymphocyte ratio; WBC, white blood cell count.

Of the 211 enrolled patients, 168 were enrolled in the training group, and 53 were enrolled in the testing group. The median overall survival time of the training group was 8 months, which was consistent with previous studies reporting that the median overall survival in advanced pancreatic cancer is approximately 6–11 months (Conroy et al., 2011; Von Hoff et al., 2013). Thus, the 8 month survival was set as the main endpoint of this work. The characteristics of the training and testing groups are listed in Table 1. The mean age of the training group was 61.05 ± 8.55 years, and that of the testing group was 61.17 ± 8.42 years (*p* > 0.05). There were 2, 42, 53, and 71 patients with stages T1–T4 disease, respectively, in the training group and 1, 10, 12, and 30 patients with stages T1–T4 disease, respectively, in the testing group (*p* > 0.05). A total of 155 (92.26%) patients were defined as M1 in the training group, and 48 (90.57%) patients were defined as M1 in the testing group (*p* > 0.05). There was no statistically significant difference in 8 month survival between these two groups (*p* = 0.581). All patients were treated with at least one dose of chemotherapy. Gemcitabine-based chemotherapy was the most common 1st-line chemotherapy regimen. There were 85.12% and 83.02% of patients who received less than third-line chemotherapy in the training group and testing group, respectively. There were no significant differences in any basic characteristics, including clinical parameters and biological parameters, between the two groups (*p* > 0.05). All continuous variables in the training and testing groups were depicted using violin plots (Figure 2).

**Table 1:** Basic characteristics of the study population.

**Variables**		**Training (*n* = 168)**	**Testing (*n* = 53)**	***p***
Age, years		61.05 ± 8.55	61.17 ± 8.42	0.928
Gender	Male	106 (63.10%)	38 (71.70%)	0.252
Main vascular invasion		71 (42.26%)	30 (56.60%)	0.068
T	T1	2 (1.19%)	1 (1.89%)	
	T2	42 (25%)	10 (18.87%)	
	T3	53 (31.55%)	12 (22.64%)	
	T4	71 (42.26%)	30 (56.60%)	0.297
N	N0	29 (17.26%)	7 (13.21%)	
	N1	139 (82.74%)	46 (86.79%)	0.486
M	M0	13 (7.74%)	5 (9.43%)	
	M1	155 (92.26%)	48 (90.57%)	0.694
Retroperitoneal lymph node metastasis		95 (56.55%)	31 (58.49%)	0.803
Liver metastasis		106 (63.10%)	36 (67.92%)	0.522
Lung metastasis		19 (11.31%)	5 (9.43%)	0.702
Peritoneal metastasis		21 (12.5%)	7 (13.21%)	0.893
Ascites		21 (12.5%)	4 (7.55%)	0.456
Size of the largest tumor of pancreas, cm		4.61 ± 1.67	4.94 ± 2.02	0.237
Tumor position of pancreas	Head and/or neck	66 (39.29%)	23 (43.40%)	
	Body and/or tail	102 (60.71%)	30 (56.60%)	0.595
Stomach invasion		60 (35.71%)	15 (28.30%)	0.406
Duodenum invasion		22 (13.10%)	9 (16.98%)	0.478
Liver metastasis number	<6	88 (52.38%)	27 (50.94%)	
	≥6	80 (47.62%)	26 (49.06%)	0.855
Size of the largest tumor of liver, cm		1.51 ± 1.85	1.89 ± 2.25	0.217
CEA, ng/mL (log-value)		0.96 ± 0.71	1.15 ± 0.77	0.103
CA199, U/mL (log-value)		2.96 ± 1.35	2.99 ± 1.10	0.865
Albumin/globin		1.63 ± 0.36	1.64 ± 0.37	0.855
ALT, U/L (log-value)		1.33 ± 0.32	1.32 ± 0.33	0.813
AST, U/L (log-value)		1.38 ± 0.24	1.34 ± 0.21	0.283
Creatinine, umol/L		63.81 ± 14.65	67.34 ± 16.48	0.141
Total bilirubin, umol/L (log-value)		1.12 ± 0.26	1.11 ± 0.27	0.849
Direct bilirubin, umol/L (log-value)		0.71 ± 0.34	0.73 ± 0.34	0.599
Indirect bilirubin, umol/L (log-value)		0.88 ± 0.24	0.84 ± 0.27	0.335
Hemoglobin, g/L (log-value)		2.09 ± 0.06	2.11 ± 0.06	0.066
Neutrophil/lymphocyte (log-value)		0.52 ± 0.26	0.57 ± 0.26	0.227
Platelet/lymphocyte (log-value)		2.14 ± 0.22	2.15 ± 0.19	0.622
WBC, 10^9^/L (log-value)		0.78 ± 0.17	0.83 ± 0.16	0.079
HBV		12 (7.14%)	3 (5.67%)	0.724
Palliative 1st line protocol	FOLFIRINOX	13 (7.74%)	4 (7.55%)	
	Gemcitabine-based chemotherapy	151 (89.88%)	49 (92.45%)	
	Others	4 (2.38%)	0 (0%)	0.524
Chemotherapy beyond 1st line protocol	<3rd line palliative chemotherapy	143 (85.12%)	44 (83.02%)	
	≥3rd line palliative chemotherapy	25 (14.88%)	9 (16.98%)	0.712
Overall survival >8 months		91 (54.17%)	31 (58.49%)	0.581

*CEA, carcinoembryonic antigen; CA199, carbohydrate antigen 199; ALT, alanine transaminase; AST, aspartate transaminase; WBC, white blood cell; HBV, hepatitis B virus*.

### Prognostic Factors for 8 Month Survival

**Figure 3:**
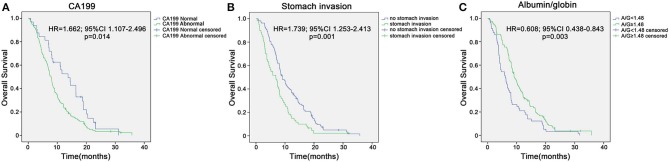
Kaplan–Meier overall survival curves for the patients with unresectable pancreatic cancer in the training sample of 168 patients. **(A)** Overall survival of patients with abnormal CA199 decreased significantly compared with that of patients with normal CA199 (median survival, 7.80 vs. 13.73 months, *p* < 0.05). **(B)** Overall survival of patients with stomach invasion decreased significantly compared with that of patients with no stomach invasion (median survival, 6.83 vs. 9.10 months, *p* < 0.05). **(C)** Overall survival of patients with low AGR decreased significantly compared with that of patients with high AGR (median survival, 6.10 vs. 9.10 months, *p* < 0.05).

In the training group of 168 patients, liver metastasis (HR 0.51, *p* = 0.041), stomach invasion (HR 0.408, *p* = 0.007), size of the largest tumor of the liver (HR 0.778, *p* = 0.008), CA199 (HR 0.685, *p* = 0.002), AGR (HR 2.885, *p* = 0.002), WBC (HR 0.092, *p* = 0.016), and gemcitabine-based chemotherapy as the first-line therapy (HR 7.401, *p* = 0.009) were related to 8 month survival in the univariate analysis (Table 2). ROC curve analysis was applied to categorize the optimal cutoff value of the AGR for 8 month survival, which was set as 1.48. We classified the patients into groups of ‘high AGR (≥1.48)' and ‘low AGR (<1.48)'. These seven variables were selected as potential independent risk factors in the multivariate analysis. The multivariate logistic regression confirmed stomach invasion (HR 0.473, *p* = 0.04), CA199 (HR 0.754, *p* = 0.046), and AGR (HR 2.360, *p* = 0.026) as independent predictors of 8 month survival (Table 2). In the training group of 168 patients, the Kaplan–Meier curve indicated that the OS of patients with abnormal CA199 (median survival, 7.80 vs. 13.73 months, *p* < 0.05), stomach invasion (median survival, 6.83 vs. 9.10 months, *p* < 0.05) and low AGR (median survival, 6.10 vs. 9.10 months, *p* < 0.05) decreased significantly (Figures 3A–C).

**Table 2:** Univariate and multivariate analyses of clinical characteristics associated with 8 month survival of the training group of 168 patients.

		**Univariate analysis**			**Multivariate analysis**		
Variables		HR	95% C.I.	*p*	HR	95% C.I.	*p*
Age, years		0.986	0.951–1.022	0.432			
Gender	Female	1					
	Male	0.702	0.372–1.323	0.274			
Main vascular invasion		1.419	0.764–2.633	0.268			
T	T1–T2	1					
	T3–T4	1.058	0.523–2.14	0.876			
N	N0	1					
	N1	0.566	0.245–1.303	0.181			
M	M0	1					
	M1	0.328	0.087–1.239	0.1			
Retroperitoneal lymph node metastasis		0.713	0.385–1.319	0.281			
Liver metastasis		0.51	0.268–0.972	**0.041**	0.854	0.35–2.08	0.727
Lung metastasis		1.186	0.451–3.116	0.729			
Peritoneal metastasis		0.741	0.296–1.851	0.521			
Ascites		0.921	0.369–2.302	0.861			
Size of of the largest tumor of pancreas, cm		0.892	0.741–1.073	0.224			
Tumor position of pancreas	Head and/or neck	1					
	Body and/or tail	1.078	0.579–2.007	0.812			
Stomach invasion		0.408	0.214–0.779	**0.007**	0.473	0.231–0.965	**0.04**
Duodenum invasion		0.669	0.272–1.646	0.381			
Liver metastasis number	<6	1					
	≥6	0.542	0.293–1.001	0.05			
Size of of the largest tumor of liver, cm		0.778	0.645–0.938	**0.008**	0.903	0.71–1.147	0.402
CEA, ng/mL (log-value)		1.132	0.733–1.748	0.575			
CA199, U/mL (log-value)		0.685	0.536–0.875	**0.002**	0.754	0.572–0.995	**0.046**
Albumin/globin	<1.48	1					
	≥1.48	2.885	1.487–5.596	**0.002**	2.36	1.106–5.038	**0.026**
ALT, U/L (log-value)		0.76	0.293–1.968	0.572			
AST, U/L (log-value)		0.62	0.171–2.248	0.467			
Creatinine, umol/L		1.009	0.987–1.03	0.427			
Total bilirubin, umol/L (log-value)		3.133	0.888–11.063	0.076			
Direct bilirubin, umol/L (log-value)		1.624	0.643–4.104	0.305			
Indirect bilirubin, umol/L (log-value)		3.81	0.996–14.578	0.051			
Hemoglobin, g/L (log-value)		0.099	0.001–13.453	0.356			
Neutrophil/lymphocyte (log-value)		0.409	0.121–1.378	0.149			
Platelet/lymphocyte (log-value)		0.818	0.2–3.346	0.78			
WBC, 10^9^/L (log-value)		0.092	0.013–0.644	**0.016**	0.369	0.043–3.168	0.363
HBV		0.845	0.261–2.737	0.779			
Gemcitabine-based chemotherapy in 1st line		7.401	1.636–33.487	**0.009**	3.768	0.753–18.865	0.107

*CEA, carcinoembryonic antigen; CA199, carbohydrate antigen 199; ALT, alanine transaminase; AST, aspartate transaminase; WBC, white blood cell; HBV, hepatitis B virus*.

*p-value < 0.05 are indicated in bold*.

### Artificial Neural Network Models and Logistic Regression Models

**Figure 4:**
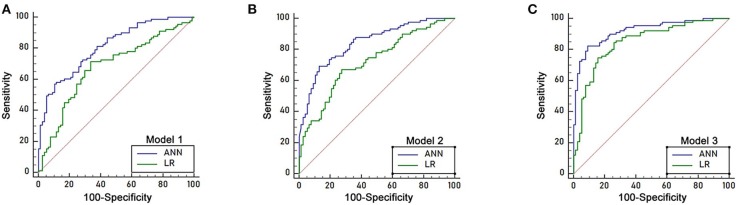
ROC curve of the logistic regression models and ANN models in the training sample of 168 patients. **(A)** The area under the ROC curve (AUC) of ANN model 1 was 0.811 (95% C.I. = 0.743–0.867), which was higher than that of LR model 1 (AUC 0.680, 95% C.I. = 0.603–0.749, *p* < 0.05). **(B)** The area under the ROC curve (AUC) of ANN model 2 was 0.844 (95% C.I. = 0.780–0.895), which was higher than that of LR model 2 (AUC 0.722, 95% C.I. = 0.648–0.788, *p* < 0.05). **(C)** The area under the ROC curve (AUC) of ANN model 3 was 0.921 (95% C.I. = 0.869–0.957), which was higher than that of LR model 3 (AUC 0.849, 95% C.I. = 0.785–0.899, *p* < 0.05).

Three independent predictors of 8 month survival, stomach invasion, AGR and CA199, were used to build the artificial neural network and logistic regression models labeled ANN model 1 and LR model 1, respectively. The area under the ROC curve (AUC) for ANN model 1 was 0.811 (95% C.I. = 0.743–0.867), higher than that of LR model 1 with 0.680 (95% C.I. = 0.603–0.749, *p* < 0.05) (Figure 4A). We applied a cutoff of 0.559 for ANN prediction, and ANN model 1 had a sensitivity of 64.83% and a specificity of 76.62%. ANN model 1 had a higher PPV for 8 month survival prediction than that of LR model 1, reflecting the good predictive power of ANN. The PLR of the ANN model for 8 month survival prediction also remained higher than that of the LR model.

Seven predictors for 8 month survival in the univariate analysis were used to build the ANN and logistic regression models labeled ANN model 2 and LR model 2. The performance of ANN model 2 was high, with an area under the ROC curve (AUC) of 0.844(95% C.I. = 0.780–0.895), compared to that of LR model 2, with an AUC of 0.722 (95% C.I. = 0.648–0.788, *p* < 0.05) (Figure 4B). A cutoff of 0.6292 was applied for ANN prediction. ANN model 2 had a sensitivity of 69.23% and a specificity of 87.01%. The PPV and PLR for 8 month survival prediction of ANN model 2 were higher than those from LR model 2.

All 32 clinical and biological parameters were used to build ANN model 3 and LR model 3 to predict 8 month survival. The area under the ROC curve (AUC) of ANN model 3 was 0.921 (95% C.I. = 0.869–0.957), which was higher than that of LR model 3 with 0.849 (95% C.I. = 0.785–0.899, *p* < 0.05) (Figure 4C). We built three ANN models, and all these models showed that the AUC of the ANN model was higher than that of the respective LR model, with ANN model 3 having the highest performance (Table 3).

**Table 3:** Accuracy of artificial neural network and logistic regression models in the training sample of 168 patients.

	**AUC**	**95% C.I**.	**Cut-off**	**PPV**		**PLR**		**Sensitivity**	**Specificity**
				**OS ≤ 8 months**	**OS > 8 months**	**OS ≤ 8 months**	**OS > 8 months**		
ANN model 1	0.811	0.743–0.867	0.559	0.6483	0.7662	0.4589	2.7735	0.6483	0.7662
LR model 1	0.680	0.603–0.749	0.5274	0.6578	0.7065	0.44	2.037	0.6493	0.7142
*p*-value	0.0008								
ANN model 2	0.844	0.780–0.895	0.6292	0.7052	0.863	0.3536	5.3307	0.6923	0.8701
LR model 2	0.722	0.648–0.788	0.5457	0.6511	0.7439	0.4532	2.4578	0.6703	0.7272
*p*-value	0.0006								
ANN model 3	0.921	0.869–0.957	0.4122	0.8117	0.9036	0.1962	7.9326	0.8241	0.8961
LR model 3	0.849	0.785–0.899	0.5601	0.7386	0.85	0.2994	4.7948	0.7472	0.8441
*p*-value	0.03								

*ANN, artificial neural network; LR, logistic regression; PPV, positive predictive values; PLR, positive likelihood ratio. Stomach invasion, CA199, albumin/globin were used to build ANN model 1 and logistic model 1. Liver metastasis, stomach invasion, size of the largest tumor of liver, CA199, albumin/globin, white blood cell and gemcitabine-based chemotherapy in first line therapy were used to build ANN model 2 and LR model 2. All 32 characters were used to build ANN model 3 and LR model 3*.

All ANN and LR models were evaluated on the testing group of 53 patients. The accuracies of ANN model 1, ANN model 2 and ANN model 3 were 0.679, 0.698, and 0.774, respectively, which were all were higher than the accuracies of the respective LR models (0.623, 0.679, and 0.736). The k-statistics were 0.344, 0.417, and 0.527 for ANN model 1, ANN model 2, and ANN model 3 and 0.233, 0.288, and 0.434 for LR model 1, LR model 2, and LR model 3, respectively. All LR models showed a lower accuracy (Table 4).

**Table 4:** Prediction accuracy of ANN and logistic regression models in the testing group of 53 patients.

	**Model 1**		**Model 2**		**Model 3**	
	**Accuracy**	**k**	**Accuracy**	**k**	**Accuracy**	**k**
ANN	0.679	0.344	0.698	0.417	0.774	0.527
LR	0.623	0.233	0.679	0.288	0.736	0.434

*ANN, artificial neural network; LR, logistic regression*.

## Discussion

Artificial neural networks have been developed as an effective statistical technique in the last 40 years (Dayhoff and DeLeo, 2001). They have been used in many fields and established as viable computational methodologies in computer science, biochemical and medical fields (Baxt and Skora, 1996; Milik et al., 1998; Gao et al., 2019; Yin et al., 2019; Deng et al., 2020; Yu et al., 2020). The network itself consists of an input layer, one or more hidden layers, and an output layer. Compared to logistic regression, ANN applies non-linear statistics and consists of a highly interconnected set of processing units (neurons) and weighted connections; the data used to build ANN can be applied to individual cases (Naguib et al., 1998).

For the ANN model, the usual ratio of training to testing group is 7:3 or 6:2:2 (when there is a validation dataset), but the radio is not strictly controlled, as previous studies have listed 5:2:3 or 6.4: 1.6: 0.2 (Cucchetti et al., 2010; Wu et al., 2017). In our study, the data before January 2018 were used as training group, and the data after January 2018 were used to simulate external validation. In the training group (*n* = 168), 133 (80%) patients were randomly selected to train the network, while 35 (20%) for cross validation. Thus, the total ratio is 6: 1.6: 2.4 (133:35:53), which was close to 6:2:2.

Many studies have demonstrated that ANN outperformed logistic regression in predicting survival, morbidity and mortality post-surgery and cancer diagnosis accuracy (Hanai et al., 2003; Pergialiotis et al., 2018; Wise et al., 2019). However, in the field of prostate cancer, the predictive accuracy of logistic regression is better than that of ANN (Chun et al., 2007; Kawakami et al., 2008). There are few applications of ANN in pancreatic cancer, and, the applications to date have been mainly in diagnosis and differential diagnosis (Ikeda et al., 1997; Norton et al., 2001; Honda et al., 2005). Very few studies have compared the abilities of ANN and logistic regression to predict the survival of advanced pancreatic cancer patients. Except for the significant clinical variables, some researchers showed non-significant variables still play important roles in prediction (Kawakami et al., 2008; Wu et al., 2017). So, we built three ANN models with different numbers of input to compare the AUC, PPV, PLR, sensitivity, and specificity, to help with patient stratification and clinical decision making in the absence of standardized prognostic risk scores for pancreatic cancer. ANN model 1 was built based on the three independent predictive factors for 8 month survival in the multivariate analysis, ANN model 2 was built based on the seven predictive factors for 8 month survival in the univariate analysis, and ANN model 3 was built based on all thirty-two variables. This is the first study comparing ANN and logistic regression in predicting unresectable pancreatic cancer patient survival. The median OS for metastatic pancreatic cancer is approximately 6 months without systemic therapy. FOLFIRINOX offered enhanced median OS as compared to gemcitabine monotherapy (11.1 vs. 6.8 months) (Conroy et al., 2011). Gemcitabine plus nab-paclitaxel demonstrated superiority than gemcitabine with OS of 8.5 vs 6.7 months (Von Hoff et al., 2013). In our study, the median OS of the training group was 8 months, which is consistent with previous studies, so we chose 8 month survival as study's primary endpoint. The ANN models were found to be superior to linear discriminant analysis in predicting 8 month survival in the training group, and these results were further validated in the testing group. In addition, as the feature numbers increased, the prediction accuracy improved. Although ANN model 3 had the best performance, it was impractical, as 32 characters needed to be collected. Of the two rest models, ANN model 2 achieved higher accuracy than ANN model 1, and the number of characters needed to be collected were acceptable, so we recommend ANN model 2 for clinicians.

All patients included had unresectable pancreatic cancer. We collected as many clinical markers related to tumor prognosis as possible. Finally, we addressed the prognostic significance of AGR, CA199 and stomach invasion in univariate and multivariate analyses. Albumin and globulin are human serum proteins. Albumin reflects nutritional status and systemic inflammatory response in cancer patients (McMillan et al., 2001). Poor nutrition status (hypoalbuminemia) has been proven to be a negative factor of survival in multiple cancers, including hepatobiliary, lung, gastrointestinal, CNS, reproductive, and breast cancers (Onate-Ocana et al., 2007; Gupta and Lis, 2010). On the other hand, haemoglobulin plays an important role in immunity and inflammation. Chronic inflammation is considered a contributor to tumor proliferation, immune evasion and metastasis. Therefore, low albumin and high haemoglobulin may decrease the survival of cancer patients. In previous studies, the AGR has been used as a prognostic indicator in diverse human cancers (Azab et al., 2013; Lv et al., 2018). However, AGR cutoff values are diverse in different studies (Lv et al., 2018), and more accurate AGR cutoff values are expected to be found.

Tumor invasion of adjacent structures is not captured in the TNM classification of pancreatic cancer from the 8th American Joint Committee on Cancer. However, a multidisciplinary consensus group recently created a standardized language for the reporting of imaging results, and reporting the presence of extrapancreatic tumor extension was recommended (Al-Hawary et al., 2014). Stomach, as one of the adjacent structures to pancreas, were recommended to be reported present or absent of tumor involved. Stomach invasion carries the risk of haematemesis. Although the incidence of haematemesis is low, it can be life-threatening if it occurs. Additionally, according to NCCN guidelines, SBRT should not be used if invasion of the stomach is observed on imaging. These results prove that stomach invasion is a problem worthy of clinical concern. In our study, Kaplan–Meier analysis showed that overall survival decreased significantly in the stomach invasion group. To the best of our knowledge, this is the first report indicating that stomach invasion is an independent prognostic factor for the 8 month survival of advanced pancreatic cancer patients. These features deserve the doctors' attention.

Treatment option is another important factor that impacts patients' prognosis. In our study, gemcitabine-based chemotherapy as the first-line therapy (HR 7.401, *p* = 0.009) were related to 8 month survival in the univariate analysis in the training group. However, it was not confirmed in the multivariate analysis. Different from randomized clinical trial, patients' status varied in retrospective study. As there was a preference among doctors and patients to select treatment based on performance status and fitness to withstand toxicities, bias is hard to be avoided. The relative small sample size may be another reason that failed to meet the statistical significance in multivariate analysis.

In addition to selecting predictive factors for 8 month survival, we also tried to identify predictive factors for 4 month progression-free survival. Even though nine factors (liver metastasis, stomach invasion, liver metastasis number, size of the largest tumor of the liver, CA199, AGR, neutrophil/lymphocyte ratio, platelet/lymphocyte ratio, and WBC count) showed statistical significance in univariate analysis, none of them were confirmed in the multivariate analysis based on the training group data (Supplementary Table 1).

Our study had several strengths. Our study made full use of clinical data that is very convenient and easy to obtain to build models to predict the survival of patients. Our models help make more accurate predictions of OS, thus optimizing patient selection for appropriate treatment and achieving more personalized management. In addition, more accurate prediction of OS will facilitate well-balanced arms in clinical trials (Vernerey et al., 2016) and allow cross-study comparisons for research purposes. Moreover, the clinical and biological parameters in the training and testing groups were comparable (*p* > 0.05), and the testing group displayed convincing performance. However, as our models were built and tested on data that originated from one center, a multicentre study should be performed in the future to verify our findings.

## Conclusions

AGR, CA199, and stomach invasion were independent predictive factors for 8 month survival in unresectable pancreatic cancer patients. We developed convenient and reliable ANN models predicting the 8 month survival of patients with unresectable pancreatic cancer, and the validation showed superior predictive accuracy of ANN over logistic regression models. Our models may help clinicians evaluate the 8 month survival time and make appropriate recommendations for the most suitable treatment options for their patients.

## Data Availability Statement

The datasets generated for this study are available on request to the corresponding author.

## Ethics Statement

The studies involving human participants were reviewed and approved by the ethics committee of the First Affiliated Hospital, Zhejiang University School of Medicine. The patients/participants provided their written informed consent to participate in this study.

## Author Contributions

ZT wrote the manuscript. HM, JZ, BL, and XB analyzed the data. YL, XX, and CG collected the clinical and pathological information from the cancer patients. YZ and LL designed the study. WF, SD, and PZ revised the manuscript.

### Conflict of Interest

The authors declare that the research was conducted in the absence of any commercial or financial relationships that could be construed as a potential conflict of interest.

## References

[B1] Al-Hawary M. M. Francis I. R. Chari S. T. Fishman E. K. Hough D. M. Lu D. S.. (2014). Pancreatic ductal adenocarcinoma radiology reporting template: consensus statement of the society of abdominal radiology and the american pancreatic association. Gastroenterology 146, 291–304.e1. 10.1053/j.gastro.2013.11.004 24355035

[B2] Are C. Afuh C. Ravipati L. Sasson A. Ullrich F. Smith L. (2009). Preoperative nomogram to predict risk of perioperative mortality following pancreatic resections for malignancy. J. Gastrointest. Surg. 13, 2152–2162. 10.1007/s11605-009-1051-z 19806409

[B3] Azab B. Kedia S. Shah N. Vonfrolio S. Lu W. Naboush A.. (2013). The value of the pretreatment albumin/globulin ratio in predicting the long-term survival in colorectal cancer. Int. J. Colorectal Dis. 28, 1629–1636. 10.1007/s00384-013-1748-z 23857599

[B4] Baxt W. G. Skora J. (1996). Prospective validation of artificial neural network trained to identify acute myocardial infarction. Lancet 347, 12–15. 10.1016/S0140-6736(96)91555-X 8531540

[B5] Bergstra J. Bengio Y. (2012). Random search for hyper-parameter optimization. J. Mach. Learn. Res. 13, 281–305.

[B6] Bradley E. L. III. (2008). Long-term survival after pancreatoduodenectomy for ductal adenocarcinoma: the emperor has no clothes? Pancreas 37, 349–351. 10.1097/MPA.0b013e31818e9100 18953246

[B7] Braga M. Capretti G. Pecorelli N. Balzano G. Doglioni C. Ariotti R.. (2011). A prognostic score to predict major complications after pancreaticoduodenectomy. Ann. Surg. 254, 702–707; discussion 707–8. 10.1097/SLA.0b013e31823598fb 22042466

[B8] Chun F. K. Karakiewicz P. I. Briganti A. Walz J. Kattan M. W. Huland H.. (2007). A critical appraisal of logistic regression-based nomograms, artificial neural networks, classification and regression-tree models, look-up tables and risk-group stratification models for prostate cancer. BJU Int. 99, 794–800. 10.1111/j.1464-410X.2006.06694.x 17378842

[B9] Conroy T. Desseigne F. Ychou M. Bouche O. Guimbaud R. Becouarn Y.. (2011). FOLFIRINOX versus gemcitabine for metastatic pancreatic cancer. N. Engl. J. Med. 364, 1817–1825. 10.1056/NEJMoa1011923 21561347

[B10] Cress R. D. Yin D. Clarke L. Bold R. Holly E. A. (2006). Survival among patients with adenocarcinoma of the pancreas: a population-based study (United States). Cancer Causes Control 17, 403–409. 10.1007/s10552-005-0539-4 16596292

[B11] Cucchetti A. Piscaglia F. Grigioni A. D. Ravaioli M. Cescon M. Zanello M.. (2010). Preoperative prediction of hepatocellular carcinoma tumour grade and micro-vascular invasion by means of artificial neural network: a pilot study. J. Hepatol. 52, 880–888. 10.1016/j.jhep.2009.12.037 20409605

[B12] Dasari B. V. Roberts K. J. Hodson J. Stevens L. Smith A. M. Hubscher S. G.. (2016). A model to predict survival following pancreaticoduodenectomy for malignancy based on tumour site, stage and lymph node ratio. HPB 18, 332–338. 10.1016/j.hpb.2015.11.008 27037202 PMC4814610

[B13] Dayhoff J. E. DeLeo J. M. (2001). Artificial neural networks: opening the black box. Cancer 91(Suppl. 8), 1615–1635. 10.1002/1097-0142(20010415)91:8+<1615::aid-cncr1175>3.0.co;2-l 11309760

[B14] Deng S. Xiang Z. Taheri J. Mohammad K. A. Yin J. Zomaya A. (2020). Optimal application deployment in resource constrained distributed edges. IEEE Trans. Mobile Comput. 99, 1–1. 10.1109/TMC.2020.2970698

[B15] Ferlay J. Soerjomataram I. Dikshit R. Eser S. Mathers C. Rebelo M.. (2015). Cancer incidence and mortality worldwide: sources, methods and major patterns in GLOBOCAN 2012. Int. J. Cancer. 136, E359–E386. 10.1002/ijc.29210 25220842

[B16] Gao W. Zhu Y. Zhang W. Zhang K. Gao H. (2019). A hierarchical recurrent approach to predict scene graphs from a visual-attention-oriented perspective. Comput. Intell. 35, 496–516. 10.1111/coin.12202

[B17] Ghoshal U. C. Das A. (2008). Models for prediction of mortality from cirrhosis with special reference to artificial neural network: a critical review. Hepatol. Int. 2, 31–38. 10.1007/s12072-007-9026-1 19669277 PMC2716874

[B18] Gupta D. Lis C. G. (2010). Pretreatment serum albumin as a predictor of cancer survival: a systematic review of the epidemiological literature. Nutr. J. 9:69. 10.1186/1475-2891-9-69 21176210 PMC3019132

[B19] Hanai T. Yatabe Y. Nakayama Y. Takahashi T. Honda H. Mitsudomi T.. (2003). Prognostic models in patients with non-small-cell lung cancer using artificial neural networks in comparison with logistic regression. Cancer Sci. 94, 473–477. 10.1111/j.1349-7006.2003.tb01467.x 12824896 PMC11160259

[B20] Hidalgo M. Cascinu S. Kleeff J. Labianca R. Lohr J. M. Neoptolemos J.. (2015). Addressing the challenges of pancreatic cancer: future directions for improving outcomes. Pancreatology 15, 8–18. 10.1016/j.pan.2014.10.001 25547205

[B21] Honda K. Hayashida Y. Umaki T. Okusaka T. Kosuge T. Kikuchi S.. (2005). Possible detection of pancreatic cancer by plasma protein profiling. Cancer Res. 65, 10613–10622. 10.1158/0008-5472.CAN-05-1851 16288055

[B22] Ikeda M. Ito S. Ishigaki T. Yamauchi K. (1997). Evaluation of a neural network classifier for pancreatic masses based on CT findings. Comput. Med. Imaging Graph. 21, 175–183. 10.1016/S0895-6111(97)00006-2 9258595

[B23] Kawakami S. Numao N. Okubo Y. Koga F. Yamamoto S. Saito K.. (2008). Development, validation, and head-to-head comparison of logistic regression-based nomograms and artificial neural network models predicting prostate cancer on initial extended biopsy. Eur. Urol. 54, 601–611. 10.1016/j.eururo.2008.01.017 18207312

[B24] Kleeff J. Korc M. Apte M. La Vecchia C. Johnson C. D. Biankin A. V. (2016). Pancreatic cancer. Nat. Rev. Dis. Primers 2:16022 10.1038/nrdp.2016.22 27158978

[B25] Kuhlmann K. F. de Castro S. M. Wesseling J. G. ten Kate F. J. Offerhaus G. J. Busch O. R.. (2004). Surgical treatment of pancreatic adenocarcinoma; actual survival and prognostic factors in 343 patients. Eur. J. Cancer 40, 549–558. 10.1016/j.ejca.2003.10.026 14962722

[B26] Landis J. R. Koch G. G. (1977). The measurement of observer agreement for categorical data. Biometrics 33, 159–174. 10.2307/2529310 843571

[B27] Lisboa P. J. Taktak A. F. (2006). The use of artificial neural networks in decision support in cancer: a systematic review. Neural Netw. 19, 408–415. 10.1016/j.neunet.2005.10.007 16483741

[B28] Lv G. Y. An L. Sun X. D. Hu Y. L. Sun D. W. (2018). Pretreatment albumin to globulin ratio can serve as a prognostic marker in human cancers: a meta-analysis. Clin. Chim. Acta 476, 81–91. 10.1016/j.cca.2017.11.019 29170102

[B29] McMillan D. C. Watson W. S. O'Gorman P. Preston T. Scott H. R. McArdle C. S. (2001). Albumin concentrations are primarily determined by the body cell mass and the systemic inflammatory response in cancer patients with weight loss. Nutr. Cancer. 39, 210–213. 10.1207/S15327914nc392_8 11759282

[B30] Milik M. Sauer D. Brunmark A. P. Yuan L. Vitiello A. Jackson M. R.. (1998). Application of an artificial neural network to predict specific class I MHC binding peptide sequences. Nat. Biotechnol. 16, 753–756. 10.1038/nbt0898-753 9702774

[B31] Miura T. Hirano S. Nakamura T. Tanaka E. Shichinohe T. Tsuchikawa T.. (2014). A new preoperative prognostic scoring system to predict prognosis in patients with locally advanced pancreatic body cancer who undergo distal pancreatectomy with en bloc celiac axis resection: a retrospective cohort study. Surgery 155, 457–467. 10.1016/j.surg.2013.10.024 24462074

[B32] Naguib R. N. Robinson M. C. Neal D. E. Hamdy F. C. (1998). Neural network analysis of combined conventional and experimental prognostic markers in prostate cancer: a pilot study. Br. J. Cancer. 78, 246–250. 10.1038/bjc.1998.472 9683301 PMC2062883

[B33] Naito Y. Ishikawa H. Sadashima E. Okabe Y. Takahashi K. Kawahara R.. (2019). Significance of neoadjuvant chemoradiotherapy for borderline resectable pancreatic head cancer: pathological local invasion and microvessel invasion analysis. Mol. Clin. Oncol. 11, 225–233. 10.3892/mco.2019.1885 31423309 PMC6688216

[B34] Neoptolemos J. P. Kleeff J. Michl P. Costello E. Greenhalf W. Palmer D. H. (2018). Therapeutic developments in pancreatic cancer: current and future perspectives. Nat. Rev. Gastroenterol. Hepatol. 15, 333–348. 10.1038/s41575-018-0005-x 29717230

[B35] Norton I. D. Zheng Y. Wiersema M. S. Greenleaf J. Clain J. E. Dimagno E. P. (2001). Neural network analysis of EUS images to differentiate between pancreatic malignancy and pancreatitis. Gastrointest. Endosc. 54, 625–629. 10.1067/mge.2001.118644 11677484

[B36] Onate-Ocana L. F. Aiello-Crocifoglio V. Gallardo-Rincon D. Herrera-Goepfert R. Brom-Valladares R. Carrillo J. F.. (2007). Serum albumin as a significant prognostic factor for patients with gastric carcinoma. Ann. Surg. Oncol. 14, 381–389. 10.1245/s10434-006-9093-x 17160496

[B37] Penny W. Frost D. (1996). Neural networks in clinical medicine. Med. Decis. Making 16, 386–398. 10.1177/0272989X9601600409 8912300

[B38] Pergialiotis V. Pouliakis A. Parthenis C. Damaskou V. Chrelias C. Papantoniou N.. (2018). The utility of artificial neural networks and classification and regression trees for the prediction of endometrial cancer in postmenopausal women. Public Health 164, 1–6. 10.1016/j.puhe.2018.07.012 30149185

[B39] Vernerey D. Huguet F. Vienot A. Goldstein D. Paget-Bailly S. Van Laethem J. L.. (2016). Prognostic nomogram and score to predict overall survival in locally advanced untreated pancreatic cancer (PROLAP). Br. J. Cancer 115, 281–289. 10.1038/bjc.2016.212 27404456 PMC4973163

[B40] Von Hoff D. D. Ervin T. Arena F. P. Chiorean E. G. Infante J. Moore M.. (2013). Increased survival in pancreatic cancer with nab-paclitaxel plus gemcitabine. N. Engl. J. Med. 369, 1691–1703. 10.1056/NEJMoa1304369 24131140 PMC4631139

[B41] Wise E. S. Amateau S. K. Ikramuddin S. Leslie D. B. (2019). Prediction of thirty-day morbidity and mortality after laparoscopic sleeve gastrectomy: data from an artificial neural network. Surg. Endosc. 10.1007/s00464-019-07130-0. [Epub ahead of print]. 31571034

[B42] Wu C. F. Wu Y. J. Liang P. C. Wu C. H. Peng S. F. Chiu H. W. (2017). Disease-free survival assessment by artificial neural networks for hepatocellular carcinoma patients after radiofrequency ablation. J. Formosan Med. Assoc. 116, 765–773. 10.1016/j.jfma.2016.12.006 28117199

[B43] Xu J. Shi K. Q. Chen B. C. Huang Z. P. Lu F. Y. Zhou M. T. (2017). A nomogram based on preoperative inflammatory markers predicting the overall survival of pancreatic ductal adenocarcinoma. J. Gastroenterol. Hepatol. 32, 1394–1402. 10.1111/jgh.13676 27973703

[B44] Yin Y. Chen L. Xu Y. Wan J. Zhang H. Mai Z. (2019). QoS prediction for service recommendation with deep feature learning in edge computing environment. Mobile Netw. Appl. 10.1007/s11036-019-01241-7

[B45] Yu J. Zhu C. Zhang J. Huang Q. Tao D. (2020). Spatial pyramid-enhanced NetVLAD with weighted triplet loss for place recognition. IEEE Trans. Neural Netw. Learn. Syst. 31, 661–674. 10.1109/TNNLS.2019.2908982 31034423

